# Risk-Taking Behaviors Considering Internet Gaming Disorder among Iranian University Students: A Latent Class Analysis

**DOI:** 10.34172/jrhs.2022.91

**Published:** 2022-10-02

**Authors:** Faeze Ghasemi Seproo, Leila Janani, Seyed Abbas Motevalian, Abbas Abbasi-Ghahramanloo, Hamed Fattahi, Shahnaz Rimaz

**Affiliations:** ^1^Department of Epidemiology, School of Public Health, Iran University of Medical Sciences, Tehran, Iran; ^2^Department of Biostatistics, School of Public Health, Iran University of Medical Sciences, Tehran, Iran; ^3^Research Center for Addiction and Risky Behaviors (ReCARB), Psychosocial Health Research Institute (PHRI), Iran University of Medical Sciences, Tehran, Iran; ^4^Department of Public Health, School of Health, Ardabil University of Medical Sciences, Ardabil, Iran; ^5^Center for Primary Health Care Network Management, Deputy for Public Health, Iranian Ministry of Health and Medical Education, Tehran, Iran; ^6^Radiation Biology Research Center, Department of Epidemiology, School of Public Health, Iran University of Medical Sciences, Tehran, Iran

**Keywords:** Internet gaming disorder, Latent class analysis, Risk-taking behaviors, University students

## Abstract

**Background:** Dangerous behaviors adversely affect the health of adolescents and young adults. This study aimed to identify the subgroups of college students based on the parameters of risky behavior and analyze the impact of demographic factors and internet gaming disorder (IGD) belonging to each class.

**Study Design:** A cross-sectional study.

**Methods:** The study was conducted on 1355 students through a multi-stage random sampling method in 2020. A survey questionnaire was used to collect data, and all students completed 1294 sets of questionnaires. The data were analyzed using t test and latent class analysis (LCA) through SPSS and PROC LCA in SAS 9.2 software.

**Results:** Three latent classes have been identified as low-risk (75%), tobacco smoker (8%), and high-risk (17%). There was a high possibility of risky behavior in the third class. Marital status (being single) (OR = 2.28, 95% CI: 1.19-4.37), unemployment (having no job) along with education (OR = 1.56, 95% CI: 1.04-2.33), and IGD (OR = 1.06, 95% CI: 1.04-1.09) increased the risk of inclusion in the tobacco smoker class. Moreover, unemployment (having no job) along with education (OR = 1.43, 95% CI: 1.11-1.84) increased the chance of being in the high-risk class.

**Conclusion:** According to the findings of this study, 25% of the students were tobacco smokers or were in the high-risk class. The results of this study may help develop and evaluate preventive strategies that simultaneously take into account different behaviors.

## Background

 Risk-taking behaviors mean “any consciously or unconsciously regulated conduct with a perceived uncertainty about its effect or about its probable benefits or costs for one’s physical, economic or psycho-social well-being, or greater to a certain disease or ill health”.^1^ Based on this definition, unwanted pregnancies, sexually transmitted diseases, drug and alcohol abuse, injuries, and even deafness are examples of risk-taking behaviors.^2^

 Alcohol, tobacco, drug use, anti-social behavior, and sex experience at a young age are the most common problematic behaviors encountered during adolescence. According to studies, alcohol, tobacco, and other addictions at young ages lead to the use of other drugs, violent and criminal behavior, as well as physical and mental disorders.^3^ Risk-taking behaviors contribute to the major causes of mortality and disability among children and adults.^4^ The result of a study conducted on 36 European countries showed that 50% of 18-26-year-old students reported alcohol and illegal substance use.^5^ Based on the report by the USA Center for Disease Control (CDC), approximately, 50% of university students were engaged in sexual behavior.^6^

 The prevalence of most risk-taking behaviors among university students has been reported in variable-centered studies in Iran separately. Smoking rates range in Iran from 13.4% to 39.9% for male students and 0.7% to 25.5% for female ones, according to a meta-analysis.^7^ Moreover, the prevalence of substance use has been reported as 17.4% (lifetime) in Tehran and 8.3% in Tabriz.^8^ However, only a few studies have assessed the co-occurrence of risk-taking behaviors among Iranian university students.^9^

 In the past two decades, the use of the Internet and computer game playing has become common activities for adolescents and young adults.^10^ The American Psychiatric Association (APA) coined the term “internet gaming disorder (IGD)” which is described as “constant repetitive interaction with video games, typically resulting in substantial daily, job, and/or educational problems.^11^ In May 2013, IGD was conceptualized in the third section of the Diagnostic and Statistical Manual of Mental Disorders. There is a chapter on conditions for additional research 5th Edition: DSM-5.^12^ It is necessary to recognize the co-occurrence of dangerous behaviors among college students for intervention strategies. People who just take drugs, for example, may be different from those who engage in a variety of risky activities.^13^

 Latent class analysis (LCA) is defined as a statistical method that employs multivariate stratified data to experimentally assign class membership to individuals to determine the related items and relevant subgroups (latent classes).^14^ In Iran, some studies indicated the pattern and co-occurrence of risk-taking behaviors among university students^15^; however, there is little data on the impact of IGD on risk-taking behaviors.^8^ The purpose of this study was to subgroup students based on risk-taking behavior and investigate the independent role of IGD in the membership of participants in each latent class.

## Materials and Methods

 From April to July 2020, 1355 university students were entered in this cross-sectional study. The sample size was calculated with a prevalence of 17% for IGD,^16^ 0.02 degree of precision (d), and 95% confidence interval (CI). A multi-stage random sampling process was used to choose the sample from the Iran University of Medical Sciences, Tehran, Iran. All students who were undergraduate, post-graduate, professional doctorate, and Ph.D. students/candidates were eligible to participate in this study.

**Figure 1:**
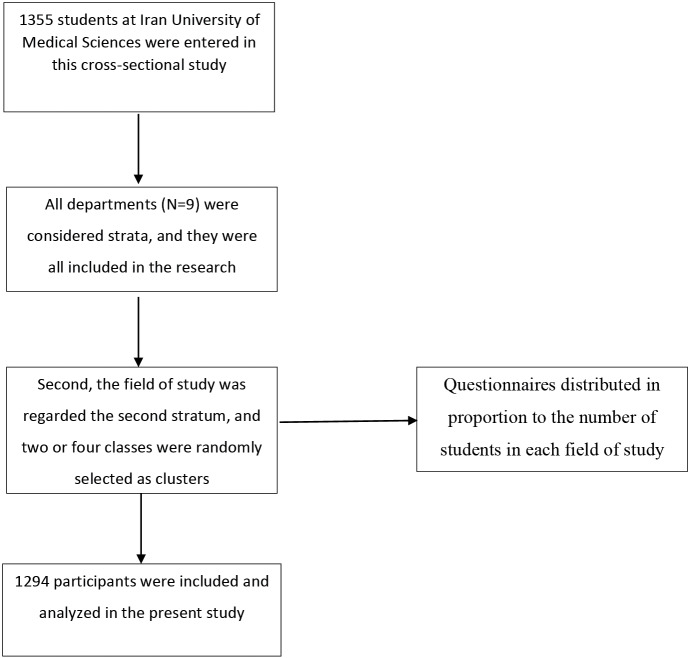
Schematic diagram of study sampling method

 To begin, all departments (N = 9) were considered strata, and they were all included in the study. Second, the field of study was regarded as the second stratum, and two or four classes were randomly picked as clusters in proportion to the number of students in each field of study. Then, students were asked to complete an electronic version of a questionnaire that was distributed on social media, such as Telegram and WhatsApp. Finally, 1294 questionnaires were completed. The schematic view of study sampling method presented in Figure 1.

 All students completed three sets of questionnaires. In the first section, we looked at demographic characteristics, such as age, gender, marital status, and the field of study. In the second section, seven dichotomous variables were used to assess risk-taking behaviors. These variables were: “self-injury”, “physical fight”, “cigarette smoking”, “hookah smoking”, “alcohol use”, “illegal drug use”, and “sexual risk behavior” in the three periods designated as lifetime, the past year, and the past month. Factors were utilized and members gave yes/no replies to them. Self-injury was measured using the direct question, “have you ever hurt yourself?”, and the physical fight was assessed using the direct question, “have you been involved in a physical fight in the last year.^17^ The information about substance use was measured using the validated questionnaire. The self-administration questionnaire used in this study was previously developed and validated by Esmaeili et al in 2017.^17^ This questionnaire was originally based on the WHO core questionnaires and WHO-ASSIST (World Health Organization-Alcohol, Smoking, and Substance Involvement Screening Test) and had been modified according to the local characteristics of substance use in Iran. We used students’ responses to the following questions to determine if they had engaged in risky sexual behavior in the past year: “Have you been engaged in heterosexual activity outside of marriage in the past year?” Next, we asked about risky sexual behaviors, including drinking drugs or alcohol before the last sex and having sex with multiple people or having sex without a condom to prevent sexually transmitted diseases.^15^ The reliability of the questionnaire for section one about risk-taking behaviors was examined twice among 45 subject students and the correlation coefficient amounted to 0.87.

 The last part of the questionnaire assessed IGD. This variable was measured using the IGDSSF 9-item questionnaire. These questions include 1. Preoccupation with Internet games, 2. Withdrawal symptoms when stop playing, 3. Tolerance (increasing playing time), 4. Failure to control attempts to participate in games, 5. Loss of interest in other activities, 6. Continued use despite awareness of the problem, 7. Family deception, 8. The mood of use, and 9. To regulate dangerous relationships or work. These questions will inquire about gaming activity over the last year. We define gaming activity as any gaming-related activity that has been played online or offline from a computer/laptop, a gaming console, or any other type of device (e.g., mobile phone, tablet, etc.). Items were rated on a scale of 1 to 5, from never at all to always. Total scores are calculated by adding all replies to all nine items, and IGDS9-SF can range from 9 to 45 points, with higher scores indicating a higher degree of IGD. In order to distinguish disordered gamers from non-disordered gamers, researchers should look for participants who have endorsed at least five of nine criteria by looking for answers like ‘5: Very Often’, which corresponds to a criterion endorsement. The validity and reliability of this questionnaire have been assessed among the Iranian population.^18^

 The LCA was used to identify risk-taking behavior groupings among students. The LCA is a categorical variable model that traditionally divides people into groups based on their shared traits. It aims to see if correlations between observable variables can be explained by latent factors in addition to measurement error.^19^ To select the best model, we calculated and compared the likelihood-ratio statistic G2, the Akaike information criterion (AIC), the Bayesian information criterion (BIC), Entropy, and the log-likelihood values across seven models. Among these indices, lower values of G2, AIC, BIC, as well as the log likelihood and higher value of Entropy showed a more optimal model fit. In addition to these indices, the interpretability and parsimony of a model could help in the selection of the final model. Classes identified in the model should be meaningful, and a simpler model is preferable. For labeling and describing the characteristics of each latent class, we used the above 0.5 item response probabilities.^19^ These variables were “self-injury”, “physical fight”, “cigarette smoking”, “hookah smoking”, “alcohol use”, “illicit drug use”, and “sexual risk”. We entered age, marital status, occupational status (having a job) along with education, IGD, and self-esteem as covariates in the LCA to finish the model. PROC LCA in SAS 9.2 software was utilized for data analysis, and *P* value < 0.05 was considered statistically significant in all analyses.

 The study protocol was approved by the Ethics Committee of Iran University of Medical Sciences, Tehran, Iran, and all students signed an informed consent form.

## Results

 The students completed 1294 out of the 1355 surveys supplied (response rate: 95%). The mean age of respondents was 19.25 ± 4.73 (range: 19-54) years. Approximately, 59% (n = 769) of participants were female, and 83% (n = 525) of the students were single. The conditional distribution of IGD scores at each level of risk-taking behavior is shown in Table 1. According to the findings, hookah usage was more common among students. In addition, the experience of self-injury has had the lowest prevalence among students at the Iran University of Medical Sciences, Tehran, Iran.

**Table 1:** Prevalence of risk-taking behaviors in a sample of students at Iran University of Medical Sciences determined by “IGD”

**Variables**	**Total, N=1294**		**Score of IGD**		***P*** ** value**
	**Number**	**Percent**	**Mean **	**SD**	
Experience of self-injury					
Yes	162	12.5	14.42	6.48	0.016
No	1132	87.5	13.11	5.65	
Physical fight					
Yes	205	15.8	12.87	5.33	0.001
No	1089	84.2	15.41	7.39	
Cigarette smoking (last year)					
Yes	298	23.0	14.50	7.14	0.001
No	996	77.0	12.91	5.25	
Hookah smoking (last year)					
Yes	311	24.0	14.11	6.88	0.010
No	983	76.0	13.01	5.36	
Alcohol use (last year)					
Yes	263	20.3	13.08	5.68	0.535
No	1031	79.7	13.32	5.80	
Illicit drug use (during lifetime)					
Yes	219	15.4	15.25	8.07	0.010
No	1075	84.6	13.11	5.51	
Sexual risky behavior (last year)					
Yes	250	19.3	13.43	6.03	0.630
No	1044	80.7	13.24	5.71	

Abbreviations: SD, standard deviation; IGD, internet gaming disorder.

 This Table also indicates that the experience of self-injury, physical fight, cigarette smoking, hookah smoking, and illicit drug use had a significant relationship with IGD. In this study, there was no significant association of alcohol use with sexual risk behavior and IGD.

Table 2 shows different indices of model selection. P-value cannot be calculated due to the high degree of freedom. Therefore, choosing the right model is judged based on G2, AIC, and BIC values. In this way, the model with lower G2, AIC, and BIC values should be selected. If the judgment was only based on the G2 value, the most suitable model will be model 7; however, the BIC value in this model was high. Therefore, a model that maintained a balance between these three values should be preferred. Entropy value can also be used. In this way, the model whose Entropy was larger was selected. Model 3 had the lowest BIC value, and there was a balance among all three values of G2, AIC, and BIC with the high value of Entropy; therefore, we decided that the three-class model was appropriate for students based on the model selection criteria and the interpretability of the results.

**Table 2:** Latent class analysis model comparison with different latent classes based on model selection statistics

**Number of latent class**	**Number of parameters estimated**	**Goodness of fit** **(G2)**	**DF**	**AIC**	**BIC**	**Entropy**	**Maximum log-likelihood**
1	7	1321.80	120	1335.80	1371.96	1.00	-4009.48
2	15	286.84	112	316.84	394.32	0.83	-3491.99
3	23	219.56	104	265.56	384.36	0.84	-3458.35
4	31	166.28	96	228.28	388.41	0.78	-3431.71
5	39	139.40	88	217.40	418.58	0.81	-3418.27
6	47	112.31	80	206.31	449.08	0.79	-3404.73
7	55	94.99	72	204.99	489.10	0.83	-3396.07

Abbreviations: AIC, Akaike information criterion; BIC, Bayesian information criterion; DF, degree of freedom.

 The LCA model’s results are presented in Table 3. The numbers in each column in Table 3 are the conditional probability that someone in a particular class responded “yes” to a certain item. These parameters are used to interpret and label the latent classes. As shown in the Table, the first category, low-risk, encompassed 75% of the students. The second class, tobacco smoker, and third class, high-risk, each accounted for 7.9% and 17.1% of the participants, respectively. The first latent class or low-risk was defined by a low likelihood of risky behaviors. In other words, the probability of occurrence of all indicators in the students of this class was low, for example, the probability of most items, such as smoking cigarettes and hookah, alcohol use, illicit drug use, sexual risk behavior, and experience of self-injury were less than 10%. However, the probability of physical fight was 13% which was the highest percentage in this class. In the second latent class or tobacco smokers, we can see a high probability of smoking cigarettes (78%) and hookah (88%), whereas in the second class, the probability of other risky items was higher than that in the first class; nonetheless, they were still less than 50%. Finally, in the third latent class, there was a high probability of responding “yes” to smoking cigarettes (80%) and hookah (62%), consuming alcohol (81%), and use of illegal drugs (55%). Because of that, this class was characterized as high-risk class. In comparison with the second class, the probability of alcohol usage was approximately 58% greater in the third class. It should be mentioned that in this class, the chance of experiencing self-injury and sexual risk behavior was relatively high.

**Table 3:** Three latent class models of risky behavior in a sample of students at Iran University of Medical Sciences

	**Latent classes**		
	**Low-risk **	**Tobacco smokers **	**High-risk**
Latent class prevalence	0.750	0.079	0.171
Item-response probabilities	**Probability of a yes response**		
Experience of self-injury	0.093	0.131	0.263
Physical fight	0.134	0.301	0.198
Cigarette smoking	**0.046**	**0.783**	**0.805**
Hookah smoking	**0.086**	**0.881**	**0.620**
Alcohol use	**0.060**	**0.230**	**0.818**
Illicit drug use	**0.010**	**0.040**	**0.551**
Sexual risk behavior	**0.042**	**0.003**	**0.457**

*Note*. The opportunity of a “No” reaction may be calculated via way of means of subtracting the item-reaction possibilities proven above from 1. Item reaction probabilities > 0.5 are shown in bold for ease of interpretation.

 We found two significant predictors of latent class membership (Table 4), implying different distribution of latent class membership across these factors. The odds of belonging to each class are compared to the reference class (low-risk class in this study) in this index. Marital status (being single) significantly increased the odds of being in tobacco smoker (OR = 2.28, 95%, CI: 1.19-4.37) class, compared to the low-risk class. Similarly, unemployment (not having a job) along with education significantly increased the odds of belonging to tobacco smoker (OR = 1.56, 95% CI: 1.04-2.33) and high-risk (OR = 1.43, 95% CI: 1.11-1.84) classes, compared to low-risk class.

**Table 4:** Predictors of belonging to a latent class of risky behavior in a sample of students at Iran University of Medical Sciences

**Predictors**	**Low-risk **	**Tobacco smokers**	**High-risk**	***P*** ** value**
	**OR (95% CI)**	**OR (95% CI)**	**OR (95% CI)**	
Age	Reference	0.99 (0.94, 1.04)	1.02 (0.99, 1.05)	0.608
Marital status (single)	Reference	2.28 (1.19, 4.37)	1.26 (0.91, 1.75)	0.024
Having a job along with education (not)	Reference	1.56 (1.04, 2.33)	1.43 (1.11, 1.84)	0.011
IGD	Reference	1.06 (1.04, 1.09)	1.02 (0.99, 1.04)	0.054
Self-esteem	Reference	0.96 (0.93, 0.99)	0.99 (0.98, 1.02)	0.462

Abbreviations: OR, odds ratio; IGD, internet gaming disorder.

## Discussion

 The result of this study showed the patterns of risk-taking behavior among participants at the Iran University of Medical Sciences, Tehran, Iran. Accordingly, three latent classes were identified as low-risk (75%), tobacco users (8%), and high-risk (17%). The probability rates of cigarette smoking and hookah smoking in the tobacco smoker class were 78% and 88%, respectively. However, in the high-risk class, the probability rates of cigarette smoking, hookah smoking, alcohol use, and illicit drug use were 80%, 62%, 81%, and 55%, respectively.

 The LCA method has been used in certain research to identify latent classes of risk-taking behaviors among university students. Researchers have utilized a variety of characteristics to identify these sub-classes, some of which will be described in more detail below.

 Chiauzzi et al. found three separated subgroups of risk-taking behaviors and drug use which were labeled as typical, high-risk, and moderately healthy.^20^ Kann et al were able to detect four classes of potential risk behaviors among female college students, including poor lifestyle but low-risk behaviors, high-risk, moderate lifestyle, but few high-risk behaviors, and health consciousness. Furthermore, in male students, the four latent classes included bad lifestyle, high-risk, moderate lifestyle, and health-conscious.^21^ Song et al reported four classes of adolescent health-risk behaviors in rural western China that include high-risk group, high level of sedentary and self-murder risk group, moderate-risk group, and low-risk group.^22^ Shekari et al discovered three sub-classes of risk-taking behaviors among students by gender in Tabriz (northwest of Iran), which were labeled as low-risk (58.9% males and 87.9% females), cigarette smoker (23.2% males and 10.6% females), and high-risk (17.9% males and 1.5% females).^8^ Another study was conducted in Bushehr province of Iran that used LCA to classify risk-taking behaviors into five categories including low-risk (79%), high-risk (7%), somewhat low-risk (6%), hookah user (4%), and very high-risk (2%).^9^

 The result of our study was similar to others^15,23,24^ regarding the prevalence of low-risk, tobacco smokers, and high-risk classes; however, it was different from that of other aforementioned studies in terms of the number of latent classes. It is worth mentioning that the studies with more classes had more variables, such as religious beliefs and lifestyle.

 The present study suggested that hookah smoking was the most common behavior among students. Availability, cheapness, the general use of hookah among families, lack of hobby for young people, and tobacco cultivation in the target population were likely to be the main reasons for a high level of hookah use in this study.^9^ In just one year (after 12 months), the lifetime prevalence of hookah usage among first-year students who were studying in Zanjan province of Iran climbed from 29% to 45%.^26^ In addition, in Iran, during lifetime, last year, and last month hookah smoking prevalence rates were reported as 26.6%, 17.8%, and 8.9%, respectively.^25^

 The probability of having sexual risk behavior was low in the low-risk class and tobacco smoker class; however, it was 45% in the high-risk class, and only a small number of students were in the third class.

 The prevalence rates of having sex outside of marriage during life and the last three months were 41.2% and 30.1%, respectively, in the United States.^26^ The low rates of extramarital sex in our study, compared to other countries, can be explained by the religious and legal prohibitions against illegal sexual practices and the cultural stigma associated with such practices in Iran.^9^ However, in comparison with the Iranian studies, this prevalence was higher. According to a study, 9.7% of Iranian university students engage in extramarital sexual activities.^9^

 Experience of self-injury had the lowest prevalence among participants (12.5%). Non-suicide self-injury is common among university students, with an estimated lifetime prevalence rate of 10.5% obtained primarily from a western sample.^27^ The result of the study showed a 12.3% lifetime prevalence of self-injury among the university sample in Iran.^28^

 An important strategy for intervention is to take into account the co-occurrence nature of risk-taking behaviors. Risk-taking behaviors have been linked in several research.^2^ Several studies have discovered a link between smoking and drug misuse, alcohol consumption and aggression, dangerous sexual activity, alcohol, and drug abuse.^29^ Levels of co-occurrence occurred in our study. In the second grade, where smoking is prevalent, hookah use is also prevalent, and in the third grade, smoking, hookah, alcohol, and drugs are prevalent, indicating that people who use tobacco and alcohol also have higher rates of drug use. In Iran, the co-occurrence of such behaviors was investigated and demonstrated in a national study.^30^ Co-occurrence of high-risk behaviors is an important issue and should be considered in prevention programs.

 The symptoms of IGD are linked to changes in lifestyle, academic inhibition, and social anxiety. Gamers with IGD may have utilized online games as a substitute for forming real-world relationships.^31^ According to the social compensation theory, online games provide a brief sensation of escape from the real world, which encourages increased game engagement.^32^ The current study found that IGD did not significantly affect students’ belonging to other classes. A study among IGD patients showed no strong statistical relationship between IGD and tobacco smoking.^18^ Furthermore, the result of a cohort study among patients seeking IGD showed that there was no significant relationship between alcohol use and IGD.^33^ Considering that this study was conducted at the beginning of the Coronavirus pandemic, the prevalence of the Coronavirus, lack of knowledge of pathogens, and exacerbation of the disease initially change the prevalence of high-risk behaviors, including smoking, hookah, alcohol, and drugs, which may have an effect on cross-sectional outbreaks of high-risk behaviors.^34^ From the real value, the closure of the universities and the quarantine of the country may increase the prevalence of using online and video games,^35^ given that in this study we used students’ responses to the high-risk behaviors and IGD questions over the past year and during the lifetime, and it seems that this pandemic has not had a considerable effect on the results of this study.

**Highlights**

- Hookah usage was more common among students.
- Three latent classes have been identified as low-risk, tobacco smoker, and high-risk.
- Unemployment (having no job) along with education significantly increased the odds of belonging to tobacco smoker and high-risk classes.

## Conclusion

 This study shows a pattern of risk-taking behaviors and some associated characteristics. The majority fell into the latent class of low-risk according to the findings of this study. Furthermore, importantly, a significant proportion of students belong to high-risk classes, and drug use in that class is relatively likely. With the recent publication of the ICD-11 and the inclusion of IGD as a disorder for the first time, there is an urgent need for interdisciplinary development of preventive approaches. Raising awareness among young people can significantly reduce associated morbidity, complications, and even death. Early control of these risky behaviors and disorders can reduce the burden of non-communicable diseases in adulthood and reduce the pressure on society and health systems.

## Acknowledgements

 This study was extracted from a postgraduate thesis in Epidemiology supported by IUMS. The authors would like to thank the Deputy of Research at IUMS.

## Authors’ contribution

 Author contributions should be clarified based on the following items:

**Conceptualization:** Ghasemi Seproo F, Janani L, Abbasi-Ghahramanloo A, Rimaz S, Motevalian SA.

**Methodology:** Janani L, Abbasi-Ghahramanloo A, Ghasemi Seproo F.

**Validation:** Ghasemi Seproo F, Fattahi H.

**Formal Analysis:** Ghasemi Seproo F, Janani L, Abbasi-Ghahramanloo A.

**Investigation:** Ghasemi Seproo F, Janani L, Abbasi-Ghahramanloo A, Rimaz S.

**Resources:** Ghasemi Seproo F, Janani L, Motevalian SA, Abbasi-Ghahramanloo A, Fattahi H, Rimaz S.

**Data Curation:** Ghasemi Seproo F, Janani L, Motevalian SA, Abbasi-Ghahramanloo A, Fattahi H, Rimaz S.

**Writing-Original Draft Preparation:** Ghasemi Seproo F.

**Writing-Review and Editing:** Abbasi-Ghahramanloo A, Fattahi H, Rimaz S.

**Supervision:** Rimaz S.

## Conflicts of interest

 The authors declare no conflict of interest in this study, and they are responsible for the content and writing the manuscript.

## Funding

 Not applicable.
